# Strategies to capitalize on cell spheroid therapeutic potential for tissue repair and disease modeling

**DOI:** 10.1038/s41536-022-00266-z

**Published:** 2022-12-09

**Authors:** Katherine H. Griffin, Shierly W. Fok, J. Kent Leach

**Affiliations:** 1 Department of Orthopaedic Surgery, UC Davis Health, Sacramento, CA 95817 USA; 2 School of Veterinary Medicine, University of California, Davis, Davis, CA 95616 USA; 3 Department of Biomedical Engineering, University of California, Davis, Davis, CA 95616 USA

**Keywords:** Regeneration, Mesenchymal stem cells

## Abstract

Cell therapies offer a tailorable, personalized treatment for use in tissue engineering to address defects arising from trauma, inefficient wound repair, or congenital malformation. However, most cell therapies have achieved limited success to date. Typically injected in solution as monodispersed cells, transplanted cells exhibit rapid cell death or insufficient retention at the site, thereby limiting their intended effects to only a few days. Spheroids, which are dense, three-dimensional (3D) aggregates of cells, enhance the beneficial effects of cell therapies by increasing and prolonging cell–cell and cell–matrix signaling. The use of spheroids is currently under investigation for many cell types. Among cells under evaluation, spheroids formed of mesenchymal stromal cells (MSCs) are particularly promising. MSC spheroids not only exhibit increased cell survival and retained differentiation, but they also secrete a potent secretome that promotes angiogenesis, reduces inflammation, and attracts endogenous host cells to promote tissue regeneration and repair. However, the clinical translation of spheroids has lagged behind promising preclinical outcomes due to hurdles in their formation, instruction, and use that have yet to be overcome. This review will describe the current state of preclinical spheroid research and highlight two key examples of spheroid use in clinically relevant disease modeling. It will highlight techniques used to instruct the phenotype and function of spheroids, describe current limitations to their use, and offer suggestions for the effective translation of cell spheroids for therapeutic treatments.

## Introduction

Cell therapy is a versatile and personalized option to address damage due to trauma, inefficient wound repair, degenerative diseases, and cancer. Current cell therapies commonly seek to stimulate tissue regeneration or replace systemic or local deficiencies in cell number or cell function. For example, erythrocyte transfusions treat anemia^1^, bone marrow transplants replace diseased marrow and regenerate hematopoietic cell lineages^2^, and chondrocyte injections are applied to treat full-thickness cartilage injuries^3^. Novel cell treatments are also used for more complex techniques, such as chimeric antigen receptor T cells (CAR-T), which target and kill tumor cells through complex immune recognition^4^. Stem and progenitor cells are used in clinical medicine, but the effective applications are limited, pertaining mostly to bone marrow transplants^5^. As in the examples above, cells are generally transplanted as individual, monodispersed cells within a suspension, most commonly by local injection. This strategy has translated to limited success due to rapid cell death upon delivery to the harsh microenvironment, insufficient retention at the site, or even damage due to shear forces associated with injection, shortening their intended effects to only a few days^6^. The loss of viability is due to limited cell–cell and cell–matrix signaling and exposure to a harsh, uncontrolled microenvironment^7–9^.

Spheroids are dense, cellular structures formed into aggregates, which are promising to increase the therapeutic potential of cell-based therapies. In order to harvest cells following culture expansion, enzymes such as trypsin are used to sever the cellular connections to the cell-secreted extracellular matrix (ECM) deposited on the culture dish. In contrast, spheroids retain their ECM, the persistence of which is critical to increase cell survival in harsh conditions and upregulate trophic factor secretion compared to dissociated cells^10,11^. Due to the presence of endogenous ECM and improved cell–cell interactions, spheroids better mimic the microenvironment found in native tissue^12,13^. The function of numerous cell types and tissues is under investigation when formed as spheroids, including hepatocytes^14^, cardiomyocytes^15^, pancreatic islet cells^16^, and mesenchymal stromal cells (MSCs), among others^17^. Though at different experimental stages, each of these has demonstrated morphologies representative of their respective tissues.

As the formation and instruction of spheroids become more precise and efficient, their clinical use will emerge as an effective treatment option for tissue repair and regeneration, wound healing, and individualized disease modeling. Preclinical studies using small and large animal models have investigated how spheroids may improve the regeneration of a multitude of organ systems, including musculoskeletal, cardiorespiratory, gastrointestinal, and endocrine systems, among others^17^. Spheroids and cellular aggregates that mimic functional native tissue frequently overlap with organoid studies, which also reveal potential uses for spheroids in clinical medicine. For example, many are using organoids and spheroids to model drug toxicity^18^ and cancer^19–21^. However, despite promising advances in these fields, common obstacles to spheroid use include limited donor supply for autologous models and the extensive time required for spheroid formation and priming.

This review will summarize the current evidence and knowledge of spheroids for use in cell-based therapies. It will describe opportunities during and after spheroid formation in which cell, signal, and biomaterial integration are combined to synergistically instruct spheroids. Finally, this review will underscore the utilization and success of in vivo models to enable translation to future clinical applications in human patients.

## Ex vivo versus in situ instruction

The behavior of cell spheroids is influenced by a multitude of factors prior to and after implantation. It is imperative to elucidate the effect of these stimuli on cell behavior to instruct their function and increase their integration with native tissue. Depending on the cell type and application, spheroids are designed to either directly or indirectly contribute to tissue formation^22,23^. The type, magnitude, and duration of cell instruction dictate whether they will play a direct anabolic role in wound healing or contribute indirectly to tissue repair through upregulated secretion of endogenous biomolecules^23^. Strategies to instruct spheroid behavior can be broadly categorized as signals applied ex vivo or in situ.

### Ex vivo instruction of spheroids

Ex vivo instruction often begins when cells are still in monolayer culture before spheroid formation. Cells can be primed by the presentation of drugs or other soluble factors, while the manipulation of microenvironmental conditions (e.g., confluency, local oxygen tension) can induce differentiation, precondition the cells for improved survival, and potentiate the desired characteristics of the target tissue. For example, MSCs are commonly cultured in osteogenic or chondrogenic media when it is desired to generate osteoblasts or chondrocytes, respectively. Additionally, hypoxic preconditioning enhances the survival of spheroids transplanted into harsh, poorly oxygenated tissue sites, resulting in increased vascularization and resultant bone formation^24^. Priming cells to enhance their differentiation or endogenous secretions is a common practice in tissue engineering and is applicable to numerous cell types.

Spheroids contribute to tissue repair through two mechanisms: (1) direct contribution to tissue formation by providing functionally differentiated cells or (2) indirectly through secretion of potent trophic factors that promote tissue formation and recruit necessary host cells for regeneration. Depending on the cell type and application, the size of the spheroid, which is dictated by cell density, will influence cell function^23^. Early studies with tumor cells and hepatocyte spheroids suggested that the beneficial effects of spheroids were largely due to the presence of a hypoxic core and the resulting oxygen gradient throughout the spheroid^25–27^. The prevailing hypothesis was that such oxygen gradients primed the cells for the harsh, ischemic in vivo environment by upregulating survival mechanisms and increasing trophic factor secretion^28^. MSC spheroids do not exhibit a hypoxic core until their density exceeds 250,000 cells (nearly 800 µm in diameter, well above the 100–200 µm diffusion limitation)^23^, but limited characterization has been reported for most other spheroid types. Larger spheroids exhibit increased apoptotic markers, perhaps due to limitations in nutrient diffusion through the spheroid. With nutrients readily available, smaller spheroids had increased metabolic activity and proliferation^23,29^, which are desirable when the function of spheroids is to directly participate in tissue formation. Where trophic factor secretion is the targeted function, spheroid diameter is determined by nutrient diffusion to balance cell viability with cell secretions. In fact, these secretions also dictate another important component of ex vivo instruction: spheroid distribution. Many have shown, largely through in vitro bioprinting techniques, that spatial distribution plays an important role in spheroid crosstalk and functional response^30–33^. For example, in a patterned microwell system, endothelial cell network formation from MSC–endothelial cell spheroids in hydrogels was most robust when separated by 200 μm in subcutaneous tissue^30^. This suggests that spheroid size and distribution are key design parameters for the critical translation of spheroids to fulfill their intended purpose.

The process of cellular self-assembly into spheroids affords an opportunity to incorporate other instructive components such as polymer nano- and microparticles, minerals, or soluble factors within each spheroid without the need for bulky biomaterials. These components are added for two purposes: (1) to present a signal to activate specific signaling pathways or modulate cell secretions or (2) to alter the critical cell–cell, cell–ECM interactions to influence cell instruction. Nano- or microparticles, either blank or loaded with bioactive molecules, can be incorporated to influence cell differentiation and secretion^34,35^. For example, microparticles of varying stiffness incorporated into MSC spheroids influence cell differentiation, and specifically, stiffer microparticles induce the osteogenic lineage and hindered adipogenesis^35^. Additionally, cell number and soluble factors, when modulated in combination with oxygen tension, induce controllable changes in MSC spheroid trophic factor secretion^36^. Others have also reported that gene therapy, when locally delivered in MSC spheroids as plasmid DNA (pDNA) in mineral-coated microparticles^37^ or siRNA in dextran microspheres^38^, effectively instructs cellular differentiation and signaling pathways. Beyond homotypic spheroids, the formation of heterotypic spheroids containing multiple cell types provides an opportunity to better mimic tissue complexity. Many different cell types are used in co-cultures depending on the desired outcome, with endothelial cells under recent examination to accelerate vascularization^39,40^. The addition of MSC-derived ECM increases spheroid responsiveness to soluble cues involved in lineage-specific differentiation^41^, and the addition of components such as nanofibers and gelatin methacryloyl (GelMA) influence spheroid size^42^ and mechanical properties^43^. Following formation, spheroids can either be directly implanted or they can undergo further instruction ex vivo.

Soluble factors are frequently used for an extended duration in culture to prime cells for transplantation. Spheroids exhibit greater osteogenic potential when differentiated as spheroids versus induction during monolayer culture followed by spheroid formation^44^. However, a potential limitation of this technique is that larger soluble cues such as growth factors do not uniformly penetrate the entire spheroid due to radial differences in cell phenotype and ECM accumulation, resulting in non-uniform differentiation. After spheroids are removed from these factors, the phenotype is lost within a few days. This limitation has been addressed by multiple groups by incorporating substrata for presenting growth factors within spheroids^45,46^. In an effort to prolong osteogenic differentiation, recombinant human bone morphogenetic protein-2 (BMP-2) was adsorbed onto hydroxyapatite (HAp) nanoparticles and incorporated into spheroids during aggregation. Under these conditions, spheroids exhibited more spatially uniform osteogenic differentiation and retained their differentiation after soluble cues were removed^46^. Others have loaded mineral-coated microparticles or MCM with adsorbed BMP-2 to drive osteogenic differentiation or microparticles loaded with TGF-β1 to promote the chondrogenic phenotype^45^. For example, mesenchymal condensates containing microparticles loaded with both TGF-*β*1 and BMP-2 undergo endochondral ossification. Though biologically distinct from spheroids, these mesenchymal condensates resulted in improved bone formation and function when combined with ambulatory mechanical loading in vivo^47^. Additionally, the incorporation of collagen and glycosaminoglycan-rich engineered cell-secreted ECM promotes and enhances MSC viability and proliferation as well as increases responsiveness to soluble cues and mechanosensitivity through Yes-associated protein and *α*2*β*1 integrin binding^41^. The incorporation of biomaterials in spheroids is a promising technique for uniform and continued spheroid instruction.

Upon injection, spheroids face many of the same challenges as monodispersed cells—limited cell viability, rapid dedifferentiation, and migration from the site of injection^10,13^. While there is evidence that injection can have a therapeutic effect, such as intramuscular injection to rescue ischemic limbs^48^, biomaterials offer a promising solution to the aforementioned problems. Indeed, the entrapment of spheroids in biomaterials is another option to further spheroid instruction. Biomaterials provide a tunable microenvironment, facilitate implantation, bridge physical defect gaps, and prolong cell viability in harsh in vivo environments^22,49^. However, the complexity of refining their characteristics to influence spheroid function is often challenging. The most promising biomaterials are biocompatible, degradable, and can be modified to mimic native ECM, which guides spheroid function and promotes integration with the surrounding environment^50^. The composition, mechanical properties, and adhesivity are of particular importance for ECM mimicry and accurate spheroid instruction. Manipulation of alginate hydrogel stiffness and availability of binding sites instructs MSC spheroid differentiation toward bone, cartilage, and fat^51^. Specifically, the modulation of polymer adhesivity by controlling Arginine-Glycine-Aspartic Acid (RGD) peptide density can regulate cell adhesion and migration from spheroids, thereby influencing bone formation^44^. Biomaterials that provide tunable in situ instruction can decrease the need for ex vivo priming and facilitate the transition to spheroid use in clinical applications.

### In situ instruction of spheroids

Spheroids injected in suspension often experience limited viability for the same reasons as dissociated cells. Unless injected into a specific anatomic space such as the joint capsule, spheroids rapidly dissociate into individual cells or dedifferentiate^13,52^. Biomaterials are a key resource in tissue engineering to localize cells at the desired delivery site and provide instructive cues to guide cell behavior. Biomaterials can be tailored to mimic the local ECM, increase cell proliferation and survival, and span gaps otherwise too large for normal and appropriate healing^53^. Incorporation of biomaterials as in situ instructive cues is also beneficial because they can decrease the amount of time in ex vivo culture, thereby delivering therapies faster and requiring fewer resources.

Modified biomaterials are under investigation to instruct undifferentiated spheroids in situ. The osteogenic potential and cell migration of MSC spheroids implanted in alginate hydrogels are mediated by the concentration of adhesive ligands in the alginate^44^. Specifically, biomaterials that provided more adhesivity were associated with increased osteogenic potential and decreased cell migration, suggesting that MSCs contained within aggregates will better maintain differentiation. Additionally, MSC spheroid differentiation induced by alginate stiffness and adhesive ligand concentration was maintained in vivo, implying that differentiation was both promoted and maintained by the tunable properties of alginate^51^. Others demonstrated that undifferentiated spheroids embedded in alginate with BMP-2 have increased osteogenic potential in femoral defects^54^. Combined, these studies indicate that modifiable biomaterials, though still under investigation, are a promising technique to guide and maintain spheroid differentiation.

## Current in vivo applications of spheroids: Cell types and target tissues

**Fig. 1:**
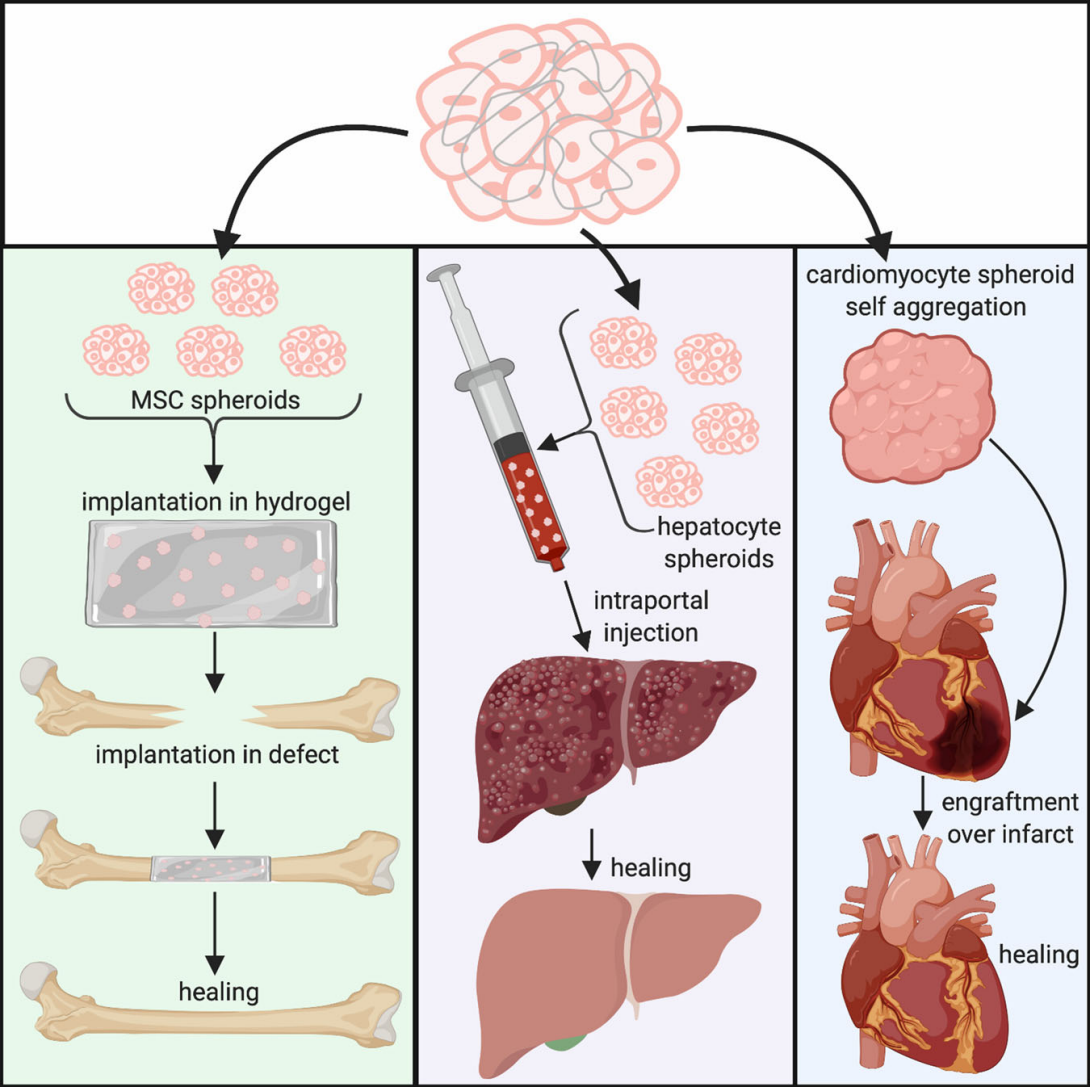
Schematic image illustrates possible therapeutic applications of cell spheroids for tissue regeneration. Examples of recent in vivo studies with spheroids: Demonstration of regenerative properties of spheroids in femoral, hepatic, and cardiac disease models using a variety of application techniques.

Spheroids are ideal building blocks for regenerative medicine due to the dense, cellular microenvironment coupled with retained cell-secreted ECM that can readily integrate with endogenous tissue. With the potential to promote tissue formation, spheroids can be used for wound healing or congenital defect corrections (Fig. 1). Below is a summary of the in vivo work to date, organized by the target tissue and application (see also, Table 1).

**Table 1:** Summary of spheroid use and application in vivo.

Target tissue		Cell types	Models tested	References
Musculoskeletal	Long bones	MSCs or ASCs Osteoblasts Chondrocytes	Segmental defects Calvarial defects Full thickness cartilage defects	^24,47,54,62,65,66,73,75,76^
	Flat bones			
	Articular cartilage			
	Dental	Dental pulp stem cells Embryonic dental cells Epithelial root sheath cells	Subcutaneous Periodontal tissue defect	^67–70^
Skin		ASCs	15 mm × 15 mm wound Skin flap	^80,81,83^
Neural		MSCs and ASCs Schwann cells Fibroblasts	Sciatic nerve gap	^85–88^
Cardiovascular	Heart	Cardiomyocytes MSCs or ASCs	Cardiac infarction Hindlimb ischemia	^48,91,93–95,98^
	Peripheral Vasculature			
Pulmonary		Adult lung cells Transbronchial lung cells	Pulmonary fibrosis	^100,101^
Digestive	Liver	Hepatocytes ASCs	Liver fibrosis Drug-induced liver injury Viral hepatitis Liver failure	^104–109^
	Intestine	Intestinal epithelial and stem cells	Functional vs. isolated loop engraftment	^113^
Endocrine	Pancreatic islets	Pancreatic islet cells	Streptozotocin-induced diabetes	^121^
	Parathyroid	Tonsil-derived MSCs	Hypoparathyroidism	^123^

### Musculoskeletal and connective tissues

#### Restoration and regeneration of mineralized tissue

In secondary bone healing, the ability of the hematoma phase to span larger, displaced fractures is sufficient to join most defects. However, nearly 10% of fractures can result in nonunion, where the gap between bone fragments is too large to span and secondary bone healing cannot occur^55^. The current gold standard of treatment is the use of an autograft, which has limited supply and associated donor site morbidity^56^. Spheroids are of particular interest in these cases since most fracture non-unions require increased angiogenesis and osteoblast activity to rebuild bone in the fracture gap^57^.

The inherent regenerative abilities of MSC spheroids alone are insufficient for healing critical-size femoral defects and require augmentation^54^. MSC spheroids induced to either the osteogenic or chondrogenic lineage can successfully repair critical-sized segmental bone defects through direct bone formation or *via* cartilage formation to follow normal healing, respectively^58^. However, if primed with only soluble cues, the osteogenic differentiation of spheroids decreases after five days upon removal of signals^13^, implying the need for better retention of spheroid phenotype. The incorporation of BMP-2, a potent osteoinductive cue, is often used to improve and maintain osteogenesis. However, the interplay between BMP-2 and implanted cells in an in vivo setting is still poorly understood^54^. Hypoxic preconditioning, a technique to prepare cells for the harsh in vivo environment, is another effective approach to increase the repair of critical-sized femoral defects with spheroids. Even without the use of soluble cues, hypoxic preconditioning can increase the osteogenic potential of MSC spheroids^24^. Chondrogenically induced MSC spheroids will stimulate bone repair *via* endochondral ossification^59–61^, but the multi-week in vitro priming remains a major limitation to these studies due to delays and challenges with maintaining sterile preparations. Recently, scaffold-free MSC condensations, which are biologically distinct from spheroids, combined with TGF-β1-loaded microspheres, successfully repaired segmental defects with in situ chondrogenic priming^47,62^, thereby increasing the potential for future clinical applications.

Unlike bones of the appendicular skeleton that form through endochondral ossification, the flat bones of the craniomaxillofacial region are formed and healed through direct intramembranous ossification. The flat bones of the skull can face many healing problems related to the lack of an intermediate cartilage callus. Cranioplasties, either achieved *via* the use of autologous bone or with biomaterials, are considered the gold standard, but feature drawbacks associated with donor site morbidity, infection, and brain swelling^63^. Recent in vivo studies demonstrate that osteogenically induced MSC spheroids increase bone formation in critically sized defects^64–66^. Allowed to self-assemble in a rotational culture system with osteogenic media, spheroids were implanted in suspension alone or with β-tricalcium phosphate (β-TCP) granules. The inclusion of β-TCP was not advantageous, as spheroid-only groups exhibited superior healing and bone regeneration after eight weeks compared to the synergistic implantation of ceramic particles and spheroids^65^. Similarly, MSC spheroids cultured in osteogenic media after formation and implanted in Matrigel scaffolds increased bone regeneration in calvarial defects after 4 weeks^66^. Neither of these studies investigated if the improved bone formation was due to the retention of the osteoblastic phenotype or the recruitment of bone-forming osteoblasts or accessory cells by trophic factor secretion. While the data clearly demonstrate that MSC spheroids can improve osteogenesis in flat bone defects, further investigation is warranted to understand the specific mechanisms responsible for intramembranous healing.

Tooth regeneration is especially challenging given the complex combination of cells required for normal tooth development. With multiple types of stem cells that differentiate into functional cells, it is difficult to distill the appropriate balance of cells and signals within cell aggregates. To address this issue, investigators have focused on a single component of the tooth^67,68^. Dental pulp stem cells formed into spheroids were instructed to closely resemble ECM found in the native pulp. When embedded in human tooth root slices, these aggregates exhibited tissue architecture similar to pulp upon subcutaneous implantation in immunodeficient mice. Endothelial cells were also added to stimulate vascularization^67^. In another example, embryonic dental cells were formed into spheroids and cultured in a semisolid state with agar added to the growth media. Upon subcutaneous implantation at the first fascial layer for 2 weeks, primitive tooth development was evident with the mineralization of enamel, as well as dentin and root formation. Additionally, one group of spheroids was co-cultured with trigeminal ganglia and demonstrated successful innervation after 2 weeks^68^. Hertwig’s epithelial root sheath cells, which are scarce, challenging to culture, and important for a tooth root and periodontium development, show enhanced survival, proliferation, and mineralization in vitro and in vivo as spheroids^69^. Atelocollagen sponges with cortical bone-derived MSC spheroids form new bone for tooth transplantation into the narrow alveolar ridge as a tooth loss solution. While the addition of MSC spheroids significantly improved bone formation, longer time points are needed for further assessment^70^. Additionally, human umbilical vein endothelial cells and MSC co-culture spheroids improved cementum formation in in vivo periodontal defect models^71^. Though far from clinical use, engineering constructs for tooth regeneration show great and versatile potential for future therapeutic applications.

#### Articular cartilage and osteochondral repair

Articular cartilage is a highly organized tissue that, once damaged, is nearly impossible to repair. The most novel cartilage treatments feature either cadaveric or non-weight-bearing hyaline cartilage grafted into the osteochondral defect, both of which feature many drawbacks including rejection and viral disease transmission, as well as limited donor supply, respectively^72^. Thus far, reports of in vivo experiments using spheroids to repair articular cartilage and osteochondral defects are sparse, though a few demonstrate promising findings. Bioprinted scaffolds with chondrogenically differentiated MSC spheroids were implanted in patellar defects and demonstrated improved cartilage formation compared to scaffolds loaded with monodispersed cells^73^. Autologous chondrocyte spheroids implanted in articular cartilage lesions resulted in “normal” or “nearly normal” macroscopic regeneration 6–72 months after implantation in 91.3% of patients^74^. Additionally, bone and cartilage were both successfully produced within the same full-thickness cartilage defect using a scaffold-free construct made with autologous MSC spheroids^75^. Each of these examples improves upon current treatments but is restricted by the time for construct differentiation and donor tissue requirements, respectively. Recently, autologous synovial MSC spheroids were implanted directly into osteochondral defects in microminipigs^76^. After 12 weeks, chondrogenesis was significantly greater in defects treated with spheroids than those untreated, and implanted MSCs were detected within the defect after one week^76^. This study demonstrates the chondrogenic potential of synovial MSCs that require minimal instruction after spheroid formation. Combined, these results reveal an increased understanding of cartilage formation and regeneration, but many limitations remain such as autologous cell source constraints and a lack of knowledge regarding the mechanical integrity of the regenerated cartilage.

While many studies have focused on regenerating cartilage and bone separately, spatially organized spheroids can recreate the complex hierarchical osteochondral tissue structure. Biphasic spheroid construct approaches mimicking the interplay between cartilage and the underlying subchondral bone increased cytokine secretion and cell–cell interaction leading to improved osteochondral repair on a critical-sized femoral trochlear groove rabbit defect. Fibrous tissue within the bone regeneration showed limitations associated with the PCL chamber, highlighting the need for further testing^77^. These findings emphasize the importance of overcoming the limitations of cartilage regeneration while studying the interplay between tissues.

#### Skin

Current treatments for non-healing skin wounds aim to promote neovascularization and epithelialization, but most are unsuccessful due to the short half-lives of active substances. MSC spheroids are under investigation due to their potent anti-inflammatory secretome, which contains some of the same factors, namely vascular endothelial growth factor (VEGF), fibroblast growth factor (FGF), and prostaglandin E_2_ (PGE_2_), used in current, acellular treatments^78,79^. The application of adipose-derived stromal cell (ASC) spheroids to wound models in vivo demonstrate increased vascularization and improved healing^80–83^. Spheroids formed on chitosan-hyaluronan membranes, which are presently used to aid wound healing, exhibit increased expression of VEGF and FGF compared to monolayer cultured ASCs. When implanted in a skin repair model, the spheroids induced increased vascularization and faster closure rates compared to dissociated ASCs^80^. However, the therapeutic benefit of ASC spheroids formed on this substrate was not compared to ASC spheroids formed in nonadhesive wells, which is one of the most common techniques. ASC spheroids were also applied to a skin flap model, which replicates a technique used in large reconstructive surgeries that suffers frequent complications due to ischemia. Spheroids demonstrated increased survival and neovascularization compared to dissociated cells when placed in this harsh environment^81^. Collectively, preclinical results are promising for the application of spheroids in skin repair. Further characterization of the MSC spheroid secretome may reveal possible improvements or alternatives for therapeutic use, such as conditioned-media constructs or upregulation of proangiogenic factors.

### Neural regeneration

Peripheral nerve autografts are the only consistently effective treatments for damaged or dying nerves^84^. Few studies have successfully enhanced peripheral nerve regeneration and bridged sciatic nerve gaps using novel spheroid techniques. One study transfected naïve MSC spheroids to overexpress brain-derived neurotrophic factor (BDNF) and loaded them into microporous poly(d,l-lactide) (PLDLA) conduits. The transfected spheroids outperformed both the naïve MSC spheroids and dissociated cells, as determined by the functional ability of the limb, myelin sheath thickness, and the time to connect nerve endings^85^. Chitosan-coated tubes filled with ASC spheroids were also investigated as a regenerative technique. Chitosan tubes loaded with spheroids resulted in significantly better healing than their acellular counterparts^86^. In both of these studies, the contribution of cells to nerve repair was not effectively demonstrated, yet this approach clearly has clinical promise for treating nerve injuries. In another example, dermal fibroblast spheroids were 3D-bioprinted into conduits to bridge a 5 mm sciatic defect^87^. Scaffold-free fibroblast constructs achieved increased regeneration compared to their silicone controls^87^. This suggests that the dense, ECM-rich microenvironment of spheroids may promote neuron growth, perhaps due to endogenous growth factors secreted by fibroblasts and presented from the ECM.

Schwann cells, responsible for the normal maintenance and regeneration of peripheral nerves, were formed into spheroids after phenotype induction from ASCs. Following implantation into a rat spinal cord injury model, local increases in myelin and neurotrophic factors promoted neuronal repair^88^. Following engraftment, the Schwann cells maintained their peripheral nervous system characteristics, even when implanted into the central nervous system environment. Despite this discrepancy, rats regained functional use of their hindlimbs. This suggests that ASC-derived Schwann cells have the potential for effective use in the regeneration of neuronal deficits. Future studies are needed to identify how these cells are contributing to nerve regeneration, either indirectly through the recruitment of endogenous cells by secreted trophic factors or through direct differentiation to cells of the neuronal lineage.

### Cardiovascular tissues

#### Heart failure and myocardial infarction

Heart failure remains the leading cause of death in the Western world^89^, and the gold standard treatment for the end-stage disease is heart transplantation. Repairing cardiac tissue during heart failure and after myocardial infarction is challenging given the finely tuned interplay between electrical stimulation and constant muscle contraction. Regenerative cell-based approaches, particularly cardiomyoplasties, have achieved limited success to date due to poor cell retention, insufficient nutrient supply, and lack of an anchoring matrix^90^. Spheroids are a promising solution as they address each of these challenges. Currently, cardiomyocyte spheroids, either 3D bioprinted^91,92^ or self-assembled^93^ into large grafts, are among the most popular in vivo applications. In preclinical studies, cardiomyocyte constructs have successfully engrafted, but improvements in both electrical signaling and angiogenesis are still warranted. Human induced pluripotent stem cell-derived cardiomyocytes, transplanted in a gelatin hydrogel, exhibited higher engraftment and angiogenesis in small and large animal cardiac cryoinjury models compared to an identical number of monodisperse cardiomyocytes^94^. In addition, co-culture spheroids of cardiomyocytes and fibroblasts or endothelial cells were secured to the outer ventricular wall and demonstrated successful engraftment one week after surgery^91,93^. However, major limitations to these studies include cell source, as both incorporated at least one cell type derived from cardiac tissue, and that engraftment was evaluated with healthy tissue, rather than diseased cardiac tissue that would be present when used clinically. Future directions should focus on trophic factor production to encourage migration of host cells into the damaged infarct, engineered materials that guide the behavior and function of cardiac spheroids, improved tissue contractility, and maintenance of wall thickness without fibrosis.

#### Peripheral vascularization

Peripheral arterial disease affects millions of people around the world, and current treatments, such as blood thinners, have limited long-term success. Cellular therapies, though attempted, have poor engraftment rates and occasionally result in more embolism events. Early spheroid studies using hindlimb ischemia models demonstrated that ASCs with upregulated hypoxia-inducible factor-1α (HIF-1α), either from hypoxic preconditioning or prolonged 3D culture, attained increased engraftment in vivo. HIF-1α improves cell viability and retention and increases proangiogenic potential, as evidenced by improved neovascularization compared to cells in monolayer^48,95^. The presentation of platelet-derived growth factor (PDGF) to ASC spheroids increased cell proliferation, endothelial differentiation, and osteogenic differentiation in vitro, as well as improved vascularization and bone regeneration in a murine calvarial defect model^96^. To further promote angiogenesis, co-culture methods with endothelial cells are also under investigation. Core-shell spheroids with a turbinate MSC and ASC core and endothelial cell shell promoted in vitro vessel-like network formation^97^. Additionally, co-cultured MSC and endothelial cell spheroids formed on hyaluronic acid hydrogels substantially improved angiogenesis when stimulated with proangiogenic growth factors such as FGF, VEGF, and PDGF^98^.

### Pulmonary tissues

#### Pulmonary fibrosis

Severe lung damage resulting from chronic bronchitis, COPD, or smoking, can result in scar tissue formation, leading to pulmonary fibrosis, an incurable, progressive disease that drastically inhibits oxygen exchange. Given the lung’s complex tissue anatomy, homotypic spheroids cannot match the numerous stem cell types found in the lung^99^. Spheroids were formed from lung explants or biopsies as a potential strategy to harness a heterogeneous cell population that spontaneously forms alveoli-like structures^100,101^. Following spheroid formation, cells were dissociated into “lung spheroid cells” (LSCs) and injected intravenously in a pulmonary fibrosis model. LSCs localized in the lung and promoted angiogenesis while also inhibiting apoptosis and fibrosis. Compared to ASCs, lungs treated with LSCs exhibited a greater reduction in fibrosis after 14 days^100^. These studies represent the potential of cells derived from experimentally formed spheroids, once dissociated, to have improved therapeutic potential than continually monodisperse or dissociated cells. The underlying cause of these improvements in cell function is currently unknown. Despite the promising regenerative capacity of LSCs, tissue source represents a fundamental limitation. To address this challenge, LSCs were formed from a transbronchial lung biopsy, a minimally invasive procedure. With a cell composition comparable to those derived from whole lung biopsies, these LSCs successfully localized to the lung following intravenous injection^101^, suggesting a strong potential for comparable regenerative ability.

### Digestive system

#### Liver

Though liver microarchitecture is highly complex, primary hepatocytes formed into spheroids will spontaneously organize into functional liver architecture^102–104^. This is of particular interest for pharmacology and toxicology in vitro studies, but there are clear regenerative applications. Following intrasplenic injection, genetically transduced human hepatocyte spheroids were successfully engrafted in uPA/SCID mouse livers, a primary model of viral hepatitis^104^. When delivered intraportally, ASC spheroids localized in the liver, increased regeneration in a liver failure model and added an immunomodulatory effect through secretions of PGE_2_^105^. Others have 3D-printed hepatocyte spheroids to form functional liver tissue with elaborate duct and sinusoid microanatomy. In these studies, constructs were metabolically functional for weeks, exhibiting appropriate glucose consumption, responsiveness to insulin, and bile acid secretion^103,106^. Following 3D printing, one study implanted the human hepatocyte constructs into the liver of nude rats and detected human albumin in the blood after one week^106^, demonstrating both survival and function of the implanted cells. Hepatic spheroid models also provide insight into potential therapeutic targets for the treatment of liver fibrosis^107,108^ and drug-induced liver injury^109^. These are important advances for using cells that are challenging to expand and maintain their phenotype under standard culture conditions.

#### Intestinal

Currently, epithelial and stem cells from the gastrointestinal tract have been formed into spheroids, but this work largely aims to use these spheroids for drug delivery, toxicity, and disease pathogenesis studies^110–112^. Limited results for in vivo studies show that functional engraftment remains elusive. For example, enteroid engraftments have only been used in bypass loops of the small intestine, implying that smooth muscle contraction or the associated flow of intestinal material may be interfering with cell function^113^.

### Endocrine

#### Pancreatic islets to treat diabetes

Pancreatic islet cell transplantation is under investigation as a curative treatment, especially for those affected by Type 1 diabetes mellitus^114,115^. However, many obstacles have inhibited successful development. As Type 1 diabetes is an autoimmune disease, implanted islet cells frequently undergo the same destruction as the patient’s original islet cells. Additionally, isolation of functional islet cells is difficult, as they are exceedingly fragile^116^. The differentiation of functional islet cells from various stem and progenitor cell populations has provided new hope for the field^117^, but it is unclear whether these cells will be immune from challenges facing other cell-based approaches. Spheroids offer a potential solution, particularly when embedded in a protective biomaterial. While this is not a novel concept^118,119^, our understanding of the immune system was so limited that little progress was made for decades. Currently, many are revisiting this potential solution for pancreatic islet transplantation with a greater appreciation for the characterization of biocompatible, non-degradable biomaterials^120^ and in conjunction with spheroids^121,122^. In mice with streptozotocin-induced diabetes, intraperitoneal implantation of collagen-alginate hydrogels loaded with pancreatic islet spheroids achieved favorable results. Normoglycemic levels (<200 mg/dL) were maintained over 4 weeks and the spheroids appropriately responded to insulin stimulation^121^. While promising, limitations to this study include the lack of longer timepoints and glucose trials upon removal of the construct.

#### Spheroids to address hypoparathyroidism

Though uncommon, primary hypoparathyroidism is a serious condition that causes dysregulation of calcium homeostasis. Aggressive calcium supplementation remains the only treatment option at this time. Cell therapies for hypoparathyroidism are limited, and in vivo studies with spheroids are even more so. The most promising study thus far demonstrated that, following removal of the parathyroid glands and subcutaneous implantation of tonsil-derived MSC spheroids, ionized calcium levels were maintained over 3 months, even in rats fed a calcium-free diet^123^.

### The MSC spheroid secretome

The MSC secretome is the collection of soluble factors and molecules secreted into the extracellular space^124^. Many hypothesize that the regenerative effects of MSCs are largely due to paracrine signaling, which is upregulated in 3D culture^124^. This has led to the investigation of media conditioned by the spheroid secretome as a cell-free technique for tissue repair.

#### Secretome applications for musculoskeletal regeneration

The close association between muscle and bone is often overlooked in bone healing, but it is particularly important in comminuted and open fractures where muscle damage is prevalent^125^. Factors secreted from myoblasts (i.e., myokines) can impact healing, and myoblasts stimulated by MSC spheroids can secrete factors that drastically improve osteogenesis. To stimulate the myoblasts with the complex secretome of MSC spheroids, media was successively conditioned with MSC spheroids, then myoblasts. Alginate hydrogels containing this dual-conditioned media with or without bone marrow-derived MSCs were implanted in critically sized femoral defects. Osteogenesis was greater in groups with just media than with MSCs alone, and groups with both MSCs and conditioned media achieved the greatest bone formation^78^. Not only does this reveal the importance of muscle signaling for bone formation, but it also suggests that the MSC spheroid secretome alone can be a powerful regenerative tool. However, the crosstalk among the MSC secretome, myokines, and osteokines (osteoblast-secreted factors) requires further investigation.

MSC spheroid secretomes are also applicable to chondrogenic and chondrocyte-homing manipulation. Most progress thus far has shown great in vitro potential, with the early characterization of the proteins secreted from chondrogenically differentiated MSC spheroids^126^ and an increased ability of the MSC secretome to promote migration of chondrocytes^127^. Umbilical cord-derived MSC spheroid secretomes have been used to promote the repair of articular cartilage. Spheroid-conditioned media was injected intra-articularly in an adjuvant-induced arthritis model. Tissues treated with conditioned media had decreased symptoms of induced arthritis, as well as a slowed rate of disease progression compared to both monodispersed cells and monolayer-conditioned media^127^. It is unclear whether the secretome reduced local inflammation due to the presence of anti-inflammatory factors or if these soluble cues promoted cartilage repair. However, this approach merits further investigation to treat damaged cartilage.

#### Secretome applications for cardiovascular regeneration

Localized delivery of paracrine signals near regions of ischemia is particularly important to recruit host cells into the damaged tissue for remodeling and tissue regeneration. Based on substantial evidence for controlling the presentation of bioactive factors, biomaterials are useful for delivering soluble factors in cardiac tissue repair. The cardioprotective and proangiogenic potential of MSC spheroid-conditioned media was demonstrated in vitro in GelMA loaded with nanosilicate particles. Gels with conditioned media increased angiogenic and cardioprotective potential compared to non-loaded gels^128^. In subsequent studies, conditioned media-loaded hydrogels were injected into rat myocardium adjacent to an induced infarct. After 21 days, cardiac tissue treated with these constructs exhibited increased capillary density, decreased scar tissue, and improved cardiac function compared to hydrogel- and secretome-only groups. Additionally, no differences in inflammatory markers were detected, indicating sufficient biocompatibility of both the hydrogel and secretome components^129^. This study demonstrates the benefit of prolonged secretome presentation achieved using biomaterials. Furthermore, these studies emphasize the efficacy and benefits of cell-free approaches, particularly for tissues in which cell source and engraftment with adequate vascularization and innervation remain a limitation.

### Spheroids to model cancer

Since the 1970s, multicellular tumor cell aggregates have provided a strategy to investigate the in vivo tumor environment. Advanced characterization methods developed in the last decade have enabled new advances with spheroids as models to understand the complex 3D architecture and to test cancer therapies^130^. Many types of cancer have been modeled by spheroids, including breast^131,132^, ovarian^133,134^, prostate^135^, colorectal^136^, and bladder cancers^137^. The research topics are as vast as the cancer types investigated, ranging from mechanistic intracellular signaling characterization^138^ and genomic sequencing^139^ to understanding tumor growth rate with computer modeling^140^.

In light of a disconnect between the treatment of patient-derived xenograft (PDX) models in mice and translation to humans^141^, tumor spheroids are of particular interest to model and investigate novel cancer therapies. Highly proliferative tumors frequently outgrow their blood supply, creating a hypoxic region. As most chemotherapies are administered intravenously, they become increasingly ineffective in these hypoxic, avascular regions^142^. Facing challenges similar to conditioning spheroids with soluble factors, the penetration of drugs beyond the periphery is one of the greatest challenges in drug delivery to tumor spheroids^143^. Nanoparticle therapies are widely investigated, but thus far, clinical trials have not performed as well as in vitro data would suggest. This further highlights the need for useful, realistic 3D tumor models^144^. Different formation methods of tumor spheroids create large variances in cell behavior and drug response^145^, providing a possible explanation for the discrepancies between in vitro results and clinical outcomes. For example, MSC and osteosarcoma spheroid co-culture models have decoupled the effect of growth factor delivery on bone regeneration and tumor growth, emphasizing the importance of representative formulations for the desired applications^146^. Understanding cancer biology, particularly on an individualized basis, is integral to finding effective cancer treatments.

Pharmacologically based cancer therapies seek to balance the need for effective treatment and avoidance of drug toxicity, with the balance often insufficient to spare all patients from toxicity. In fact, toxicity is a common cancer complication, and better predictive methods of appropriate dosage are desperately needed. Cancer cell spheroids are excellent models for therapy efficacy, but spheroids from other organ systems, especially those involved in drug metabolism and excretion, are of interest for pharmacokinetics and pharmacodynamics studies to estimate individual drug tolerance levels^147^. As a complex filtration system, the kidney is important for clearing waste and metabolites from the blood. Isolated renal tubules, as aggregates, have proliferated and reorganized in vitro, displaying appropriate biochemical function, as well as anticipated response and changes to nephrotoxic drugs^148^. Acute kidney injury is a common complication of intensive drug therapies, and MSC spheroids injected into an ischemia-reperfusion injury model resulted in increased angiogenesis, decreased tissue damage, and rescued kidney function compared to monodispersed cells^149^. Hepatocyte spheroids are also investigated for drug clearance and toxicity modeling^147^. Primary hepatocyte spheroids in bioreactor cultures maintained consistent and accurate biological function for up to 4 weeks^150^. This system could easily translate to improved, individualized multi-dose drug testing. Similarly, hepatocyte spheroids bioprinted into hepatic constructs were maintained in liver-on-a-chip bioreactors for 30 days and demonstrated appropriate biological function upon insult with toxic levels of acetaminophen^151^.

### Spheroids to model infectious diseases such as COVID-19

Infectious disease studies have utilized spheroids to study pathogenic cell entry mechanisms as well as to develop spheroid-derived therapies. Some applications include tuberculosis and coronaviruses^152^. Specifically, research related to severe acute respiratory syndrome coronavirus 2 (SARS-CoV-2), commonly referred to as COVID-19, has utilized spheroids to study mechanistic details and inspire new therapies. The SARS-CoV-2 pandemic has increased the public health and economic burden, mandating an urgent need to identify the mechanisms of infection and develop effective drug therapies. 3D spheroid cultures provide a robust preclinical model to study infectious pathophysiology.

Initial research related to SARS-CoV-2 has focused on the angiotensin-converting enzyme 2 (ACE2) receptor, which was identified in 2003 as the viral mode of entry of severe acute respiratory syndrome (SARS) coronaviruses into lung epithelial cells^153^. In addition to ACE2, tyrosine-protein kinase receptor UFO (AXL) is another potential therapeutic target due to its high multi-organ expression confirmed through primary lung tissue spheroid testing and single-cell mRNA sequencing datasets^154^. Studies using primary human lung microvascular endothelial cell (HL-mECs) spheroids, which lack ACE2, have revealed the role of RGD motifs in facilitating SARS-CoV-2 infection in the absence of ACE2^155^. This highlights the role of spheroids as models in diseases such as COVID-19.

The identification of mechanistic processes associated with viral infection has allowed the further study of SARS-CoV-2 and opened possibilities for novel therapeutic design. Spheroids have inspired and are key components of the nanodecoy technology designed to decrease viral load. Nanodecoys are homogeneous 320 nm-sized nanovesicles expressing membranous ACE2. Lung spheroids composed of a mixture of mesenchymal cells and lung epithelial cells with type I and II pneumocytes undergo serial extrusion to produce ACE2-presenting nanovesicles. Nanovesicles are delivered through nebulization, neutralize viral infection by presenting membranous target receptors and peptides utilized by SARS-CoV-2, and are cleared by macrophages 72 h after delivery^156^. Advances in this area showcase the role of spheroids as preclinical models and tools for a variety of applications including the development of novel therapies.

## Clinical future of spheroids

Spheroids possess great therapeutic potential through direct cell differentiation to serve as building blocks for repairing tissue, or through more subtle methods such as increased trophic factor secretions to recruit cells and promote vascularization. However, applications of spheroids have primarily been studied in model systems. As dense, engineered aggregates, the suitability of spheroids for all tissue types is not established. For example, native lung tissue is not composed of dense cell aggregates, and excessive ECM can result in fibrotic pathologies^157^. Clearly, many obstacles related to the spheroid formation and their application remain.

Current limitations to clinical spheroid use include cell source, of which there are two broad categories: allogeneic or autologous. Allogeneic cells are preferred when large numbers of cells are necessary and immune responses are appropriately managed. The use of allogeneic cells is most appropriate if extensive ex vivo instruction continues, allowing for time-consuming construct preparation. Similar to blood bank analyses of blood donations, allogeneic cell collections could be well characterized, differentiated, and sorted into designated clinical applications based on their differentiation and regeneration potential. However, the possibility of construct rejection, even with careful cross-matching, is high, and the economic burden associated with ex vivo priming is potentially cost-prohibitive on a large scale^158^. On the other hand, autologous cell sources face many of the same limitations as current cell therapies, such as limited donor supply and donor site pain and morbidity, as well as the increased time and costs associated with ex vivo instruction. Nevertheless, improvements in cell collection, spheroid formation, and in situ instruction could make a critical difference, enabling the use of autologous cells as a source for spheroids in the clinic.

Cell-free therapies represent another approach to address challenges in cell sourcing through the use of spheroid-conditioned media. As previously stated, the secretomes of many types of spheroids are under investigation, and conditioned media alone can promote tissue regeneration^78,124,126–129^. Beyond trophic factor secretion in the secretome, extracellular vesicles (EVs) including apoptotic bodies, microparticles, and exosomes, also play a role in extracellular signaling. Much like the rest of the secretome, exosomes are upregulated in spheroids compared to cells in monolayer^159^. Additionally, the characterization of neuro-stimulated MSC spheroid EVs has shown immunomodulatory, angiogenic, and neurogenic cytokine and micro-RNA inclusions. When applied to in vitro models, these EVs effectively stimulated angiogenic and neurogenic differentiation in appropriate cell lines^160^. Though preclinical data are limited, extracellular signals from spheroids may prove just as potent as the cells themselves.

The use of more accurate model systems is a key challenge that must be addressed to interrogate the contribution of spheroids and propel them to the clinic. Current models provide useful insights into the function and regenerative capacity of spheroids, yet they fail to account for age and concurrent disease states. These are highly relevant factors that apply to most of the human population that these therapies aim to target. In vivo studies are usually performed in healthy, young adult animals, whereas the populations seeking regenerative therapeutics are typically the elderly affected by degenerative processes and comorbidities or children with congenital defects. Healing in both populations is vastly different from a young adult, but spheroid investigations accounting for these differences are scarce.

Veterinary medicine, along with small and large animal models, can provide improved models to study many of these challenges. Promising results in small and large animals can motivate the need for large-scale studies, better representing the magnitude required in humans. Additionally, many naturally occurring disease processes in animals provide excellent models of human disease. Such diseases are often accelerated, equally complex, and in some cases, managed similarly clinically. For example, canine osteosarcoma (OS) is similar to OS in humans. Commonly affecting the long bones and metastasizing to the lungs, canine OS presents similarly and follows a comparable disease pattern, yet the disease is significantly accelerated. Such models can then serve as an improved platform to not only study the pathogenesis of the disease but also test potential treatment strategies.

**Fig. 2:**
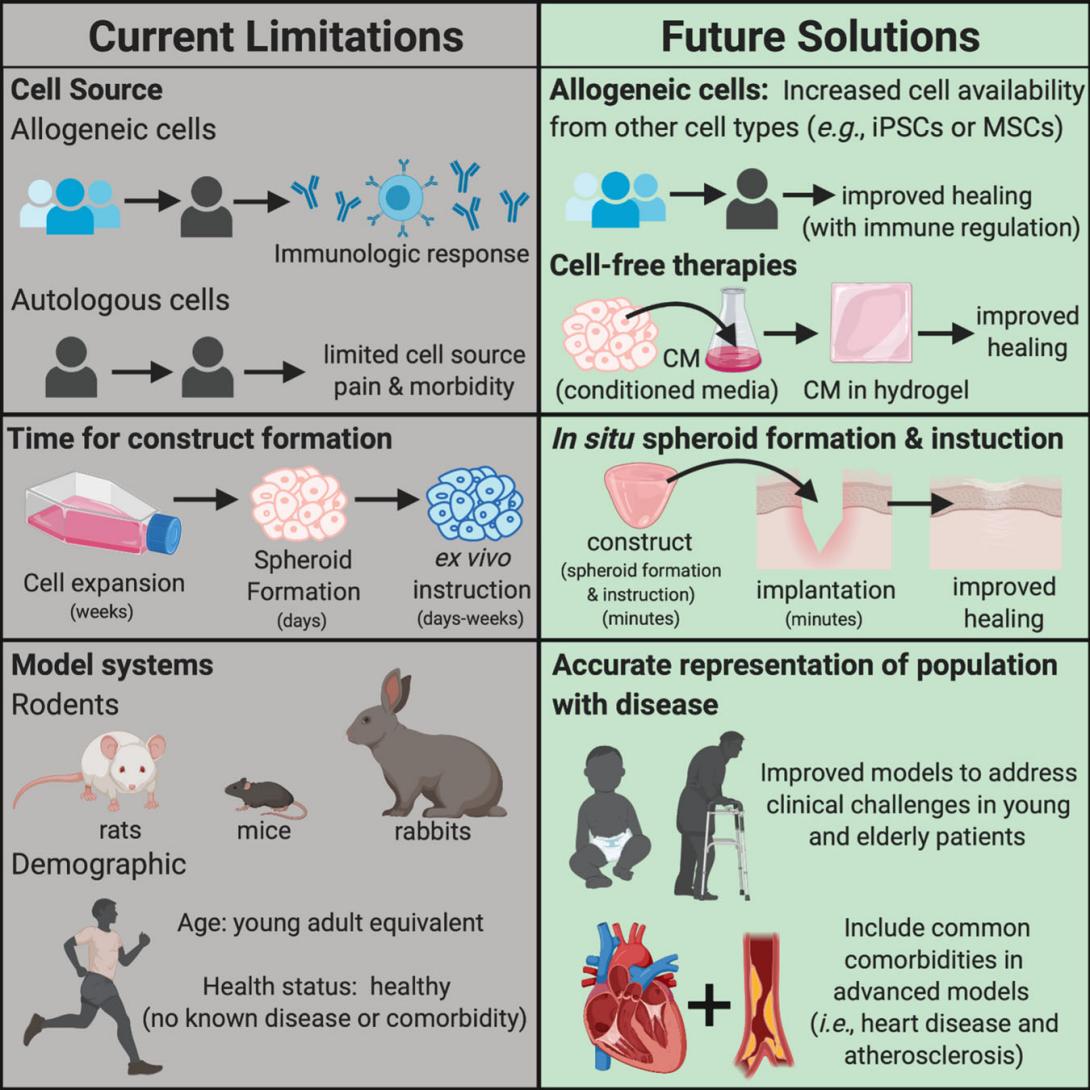
Schematic image illustrates the limitations and future solutions for spheroid use in therapeutic applications. Current limitations to clinical spheroid use include reliable and safe cell sources, temporal factors in the generation, and translation between model systems and human patients.

This review highlights the current state of spheroids for clinical use and demonstrates their immense capacity for tissue regeneration (Fig. 2). Their relevance to future clinical medicine will be determined by the successful development of methods to accelerate spheroid formation, reliable in situ instruction, and characterization, control, and utilization of their secreted factors. By establishing these techniques, spheroids may become an advanced cell therapy for regenerative medicine.

## Data Availability

Authors can confirm that all relevant data are included in the article and/or its supplementary information files

## References

[1] Liumbruno G, Bennardello F, Lattanzio A, Piccoli P, Rossetti G (2009). Recommendations for the transfusion of red blood cells. Blood Transfus..

[2] Majhail NS (2015). Indications for autologous and allogeneic hematopoietic cell transplantation: guidelines from the American Society for Blood and Marrow Transplantation. Biol. Blood Marrow Transpl..

[3] Mistry H (2017). Autologous chondrocyte implantation in the knee: systematic review and economic evaluation. Health Technol. Assess..

[4] Miliotou AN, Papadopoulou LC (2018). CAR T-cell therapy: a new era in cancer immunotherapy. Curr. Pharm. Biotechnol..

[5] Copelan EA (2006). Hematopoietic stem-cell transplantation. N. Engl. J. Med..

[6] Baldari S (2017). Challenges and strategies for improving the regenerative effects of mesenchymal stromal cell-based therapies. Int. J. Mol. Sci..

[7] Moya A (2018). Human mesenchymal stem cell failure to adapt to glucose shortage and rapidly use intracellular energy reserves through glycolysis explains poor cell survival after implantation. Stem Cells.

[8] Manassero M (2016). Comparison of survival and osteogenic ability of human mesenchymal stem cells in orthotopic and ectopic sites in mice. Tissue Eng. Part A.

[9] Zhang M (2001). Cardiomyocyte grafting for cardiac repair: graft cell death and anti-death strategies. J. Mol. Cell. Cardiol..

[10] Hoch AI (2016). Cell-secreted matrices perpetuate the bone-forming phenotype of differentiated mesenchymal stem cells. Biomaterials.

[11] Laschke MW, Menger MD (2017). Life is 3D: boosting spheroid function for tissue engineering. Trends Biotechnol..

[12] Harvestine JN (2016). Extracellular matrix-coated composite scaffolds promote mesenchymal stem cell persistence and osteogenesis. Biomacromolecules.

[13] Murphy KC, Hoch AI, Harvestine JN, Zhou D, Leach JK (2016). Mesenchymal stem cell spheroids retain osteogenic phenotype through alpha2beta1 signaling. Stem Cells Transl. Med..

[14] Bell CC (2016). Characterization of primary human hepatocyte spheroids as a model system for drug-induced liver injury, liver function and disease. Sci. Rep.-Uk.

[15] Mattapally S (2018). Spheroids of cardiomyocytes derived from human-induced pluripotent stem cells improve recovery from myocardial injury in mice. Am. J. Physiol. Heart Circ. Physiol..

[16] Jo YH (2014). Artificial islets from hybrid spheroids of three pancreatic cell lines. Transpl. Proc..

[17] Ong CS (2018). In vivo therapeutic applications of cell spheroids. Biotechnol. Adv..

[18] Shen, J. X., Youhanna, S., Shafagh, R. Z., Kele, J. & Lauschke, V. M., Organotypic and microphysiological models of liver, gut and kidney for studies of drug metabolism, pharmacokinetics and toxicity. *Chem. Res. Toxicol*. **33**, 38–60 (2019). 10.1021/acs.chemrestox.9b00245 31576743

[19] Weiswald LB, Bellet D, Dangles-Marie V (2015). Spherical cancer models in tumor biology. Neoplasia.

[20] Kim JB, Stein R, O’Hare MJ (2004). Three-dimensional in vitro tissue culture models of breast cancer—a review. Breast Cancer Res. Treat..

[21] Rodrigues T (2018). Emerging tumor spheroids technologies for 3D in vitro cancer modeling. Pharm. Ther..

[22] Gonzalez-Fernandez T, Sikorski P, Leach JK (2019). Bio-instructive materials for musculoskeletal regeneration. Acta Biomater..

[23] Murphy KC (2017). Measurement of oxygen tension within mesenchymal stem cell spheroids. J. R. Soc. Interface.

[24] Ho SS, Hung BP, Heyrani N, Lee MA, Leach JK (2018). Hypoxic preconditioning of mesenchymal stem cells with subsequent spheroid formation accelerates repair of segmental bone defects. Stem Cells.

[25] Franko AJ, Sutherland RM (1979). Oxygen diffusion distance and development of necrosis in multicell spheroids. Radiat. Res..

[26] Chen B, Longtine MS, Nelson DM (2013). Pericellular oxygen concentration of cultured primary human trophoblasts. Placenta.

[27] Mueller-Klieser W (1984). Method for the determination of oxygen consumption rates and diffusion coefficients in multicellular spheroids. Biophys. J..

[28] Korff T, Augustin HG (1998). Integration of endothelial cells in multicellular spheroids prevents apoptosis and induces differentiation. J. Cell Biol..

[29] Curcio E (2007). Mass transfer and metabolic reactions in hepatocyte spheroids cultured in rotating wall gas-permeable membrane system. Biomaterials.

[30] Kim SJ (2022). Spatially arranged encapsulation of stem cell spheroids within hydrogels for the regulation of spheroid fusion and cell migration. Acta Biomater..

[31] Daly AC, Davidson MD, Burdick JA (2021). 3D bioprinting of high cell-density heterogeneous tissue models through spheroid fusion within self-healing hydrogels. Nat. Commun..

[32] Ayan B, Wu Y, Karuppagounder V, Kamal F, Ozbolat IT (2020). Aspiration-assisted bioprinting of the osteochondral interface. Sci. Rep..

[33] Ayan B (2020). Aspiration-assisted bioprinting for precise positioning of biologics. Sci. Adv..

[34] Ankrum JA (2014). Engineering cells with intracellular agent-loaded microparticles to control cell phenotype. Nat. Protoc..

[35] Abbasi F, Ghanian MH, Baharvand H, Vahidi B, Eslaminejad MB (2018). Engineering mesenchymal stem cell spheroids by incorporation of mechanoregulator microparticles. J. Mech. Behav. Biomed. Mater..

[36] Murphy KC (2017). Multifactorial experimental design to optimize the anti-inflammatory and proangiogenic potential of mesenchymal stem cell spheroids. Stem Cells.

[37] Khalil AS, Yu X, Dang PN, Alsberg E, Murphy WL (2019). A microparticle approach for non-viral gene delivery within 3D human mesenchymal stromal cell aggregates. Acta Biomater..

[38] McMillan A (2021). Hydrogel microspheres for spatiotemporally controlled delivery of RNA and silencing gene expression within scaffold-free tissue engineered constructs. Acta Biomater..

[39] Sanchez-Palencia DM, Bigger-Allen A, Saint-Geniez M, Arboleda-Velasquez JF, D’Amore PA (2016). Coculture assays for endothelial cells-mural cells interactions. Methods Mol. Biol..

[40] Vorwald CE, Murphy KC, Leach JK (2018). Restoring vasculogenic potential of endothelial cells from diabetic patients through spheroid formation. Cell. Mol. Bioeng..

[41] Gonzalez-Fernandez T, Tenorio AJ, Saiz AM, Leach JK (2022). Engineered cell-secreted extracellular matrix modulates cell spheroid mechanosensing and amplifies their response to inductive cues for the formation of mineralized tissues. Adv. Healthc. Mater..

[42] Lee J, Lee S, Kim SM, Shin H (2021). Size-controlled human adipose-derived stem cell spheroids hybridized with single-segmented nanofibers and their effect on viability and stem cell differentiation. Biomater. Res..

[43] Kim EM (2022). Effects of mechanical properties of gelatin methacryloyl hydrogels on encapsulated stem cell spheroids for 3D tissue engineering. Int. J. Biol. Macromol..

[44] Ho SS, Keown AT, Addison B, Leach JK (2017). Cell migration and bone formation from mesenchymal stem cell spheroids in alginate hydrogels are regulated by adhesive ligand density. Biomacromolecules.

[45] Dang PN (2016). Controlled dual growth factor delivery from microparticles incorporated within human bone marrow-derived mesenchymal stem cell aggregates for enhanced bone tissue engineering via endochondral ossification. Stem Cell Transl. Med..

[46] Whitehead J, Kothambawala A, Kent Leach J (2019). Morphogen delivery by osteoconductive nanoparticles instructs stromal cell spheroid phenotype. Adv. Biosyst..

[47] Herberg S (2019). Combinatorial morphogenetic and mechanical cues to mimic bone development for defect repair. Sci. Adv..

[48] Bhang SH (2011). Angiogenesis in ischemic tissue produced by spheroid grafting of human adipose-derived stromal cells. Biomaterials.

[49] Gionet-Gonzales MA, Leach JK (2018). Engineering principles for guiding spheroid function in the regeneration of bone, cartilage, and skin. Biomed. Mater..

[50] Marin E, Boschetto F, Pezzotti G (2020). Biomaterials and biocompatibility: an historical overview. J. Biomed. Mater. Res. A.

[51] Hung BP (2019). Defining hydrogel properties to instruct lineage- and cell-specific mesenchymal differentiation. Biomaterials.

[52] Uth K, Trifonov D (2014). Stem cell application for osteoarthritis in the knee joint: a minireview. World J. Stem Cells.

[53] Leach JK, Whitehead J (2018). Materials-directed differentiation of mesenchymal stem cells for tissue engineering and regeneration. ACS Biomater. Sci. Eng..

[54] Allen AB (2016). Environmental manipulation to promote stem cell survival in vivo: use of aggregation, oxygen carrier, and BMP-2 co-delivery strategies. J. Mater. Chem. B.

[55] Mills LA, Aitken SA, Simpson A (2017). The risk of non-union per fracture: current myths and revised figures from a population of over 4 million adults. Acta Orthop..

[56] Conway JD (2010). Autograft and nonunions: morbidity with intramedullary bone graft versus iliac crest bone graft. Orthop. Clin. North Am..

[57] Schlundt C (2018). Clinical and research approaches to treat non-union fracture. Curr. Osteoporos. Rep..

[58] Verrier S (2016). Tissue engineering and regenerative approaches to improving the healing of large bone defects. Eur. Cells Mater..

[59] Sheehy EJ, Vinardell T, Buckley CT, Kelly DJ (2013). Engineering osteochondral constructs through spatial regulation of endochondral ossification. Acta Biomater..

[60] Farrell E (2011). In-vivo generation of bone via endochondral ossification by in-vitro chondrogenic priming of adult human and rat mesenchymal stem cells. BMC Musculoskelet. Disord..

[61] Pelttari K (2006). Premature induction of hypertrophy during in vitro chondrogenesis of human mesenchymal stem cells correlates with calcification and vascular invasion after ectopic transplantation in SCID mice. Arthritis Rheum..

[62] McDermott, A. M. et al. Recapitulating bone development through engineered mesenchymal condensations and mechanical cues for tissue regeneration. *Sci. Transl. Med*. **11**, eaav7756 (2019). 10.1126/scitranslmed.aav7756 PMC6959418 31167930

[63] Aydin S, Kucukyuruk B, Abuzayed B, Aydin S, Sanus GZ (2011). Cranioplasty: Review of materials and techniques. J. Neurosci. Rural Pract..

[64] Whitehead J (2021). Hydrogel mechanics are a key driver of bone formation by mesenchymal stromal cell spheroids. Biomaterials.

[65] Suenaga, H., Furukawa, K. S., Suzuki, Y., Takato, T. & Ushida, T. Bone regeneration in calvarial defects in a rat model by implantation of human bone marrow-derived mesenchymal stromal cell spheroids. *J. Mater. Sci.-Mater. Med.* **26**, 254 (2015). 10.1007/s10856-015-5591-3 PMC4598349 26449444

[66] Yamaguchi Y, Ohno J, Sato A, Kido H, Fukushima T (2014). Mesenchymal stem cell spheroids exhibit enhanced in-vitro and in-vivo osteoregenerative potential. BMC Biotechnol..

[67] Dissanayaka WL, Zhu L, Hargreaves KM, Jin L, Zhang C (2014). Scaffold-free prevascularized microtissue spheroids for pulp regeneration. J. Dent. Res..

[68] Kuchler-Bopp S (2016). Three-dimensional micro-culture system for tooth tissue engineering. J. Dent. Res..

[69] Duan Y (2020). Therapeutic potential of HERS spheroids in tooth regeneration. Theranostics.

[70] Sano K (2020). Co-cultured spheroids of human periodontal ligament mesenchymal stem cells and vascular endothelial cells enhance periodontal tissue regeneration. Regen. Ther..

[71] Matsumura N (2022). Tissue engineering with compact bone-derived cell spheroids enables bone formation around transplanted tooth. Tissue Eng. Regen. Med..

[72] Perera JR, Gikas PD, Bentley G (2012). The present state of treatments for articular cartilage defects in the knee. Ann. R. Coll. Surg. Engl..

[73] Huang, G. S. et al. Solid freeform-fabricated scaffolds designed to carry multicellular mesenchymal stem cell spheroids for cartilage regeneration. *Eur. Cells Mater*. **26**, 179–194; discussion 194 (2013). 10.22203/ecm.v026a13 24122653

[74] Siebold R, Karidakis G, Feil S, Fernandez F (2016). Second-look assessment after all-arthroscopic autologous chondrocyte implantation with spheroides at the knee joint. Knee Surg. Sports Traumatol. Arthrosc..

[75] Ishihara K, Nakayama K, Akieda S, Matsuda S, Iwamoto Y (2014). Simultaneous regeneration of full-thickness cartilage and subchondral bone defects in vivo using a three-dimensional scaffold-free autologous construct derived from high-density bone marrow-derived mesenchymal stem cells. J. Orthop. Surg. Res..

[76] Kondo S (2019). Transplantation of aggregates of autologous synovial mesenchymal stem cells for treatment of cartilage defects in the femoral condyle and the femoral groove in microminipigs. Am. J. Sports Med..

[77] Lee J, Lee S, Huh SJ, Kang BJ, Shin H (2022). Directed regeneration of osteochondral tissue by hierarchical assembly of spatially organized composite spheroids. Adv. Sci. (Weinh.).

[78] Saiz AM, Gionet-Gonzales MA, Lee MA, Leach JK (2019). Conditioning of myoblast secretome using mesenchymal stem/stromal cell spheroids improves bone repair. Bone.

[79] Gionet-Gonzales M (2021). Sulfated alginate hydrogels prolong the therapeutic potential of MSC spheroids by sequestering the secretome. Adv. Healthc. Mater..

[80] Hsu SH, Hsieh PS (2015). Self-assembled adult adipose-derived stem cell spheroids combined with biomaterials promote wound healing in a rat skin repair model. Wound Repair Regen..

[81] Park IS, Chung PS, Ahn JC (2016). Angiogenic synergistic effect of adipose-derived stromal cell spheroids with low-level light therapy in a model of acute skin flap ischemia. Cells Tissues Organs.

[82] Nagano H (2021). Enhanced cellular engraftment of adipose-derived mesenchymal stem cell spheroids by using nanosheets as scaffolds. Sci. Rep..

[83] Suematsu Y, Nagano H, Kiyosawa T, Takeoka S, Fujie T (2022). Angiogenic efficacy of ASC spheroids filtrated on porous nanosheets for the treatment of a diabetic skin ulcer. J. Biomed. Mater. Res. B Appl. Biomater..

[84] Faroni A, Mobasseri SA, Kingham PJ, Reid AJ (2015). Peripheral nerve regeneration: experimental strategies and future perspectives. Adv. Drug Deliv. Rev..

[85] Tseng TC, Hsu SH (2014). Substrate-mediated nanoparticle/gene delivery to MSC spheroids and their applications in peripheral nerve regeneration. Biomaterials.

[86] Hsueh YY (2014). Functional recoveries of sciatic nerve regeneration by combining chitosan-coated conduit and neurosphere cells induced from adipose-derived stem cells. Biomaterials.

[87] Yurie H (2017). The efficacy of a scaffold-free Bio 3D conduit developed from human fibroblasts on peripheral nerve regeneration in a rat sciatic nerve model. PLoS ONE.

[88] Chi GF, Kim MR, Kim DW, Jiang MH, Son Y (2010). Schwann cells differentiated from spheroid-forming cells of rat subcutaneous fat tissue myelinate axons in the spinal cord injury. Exp. Neurol..

[89] Heron, M. *Deaths: Leading Causes in 2017*. Vol. 68, 1–76 (U.S. Department of Health and Human Services, 2019).

[90] Wang F, Guan J (2010). Cellular cardiomyoplasty and cardiac tissue engineering for myocardial therapy. Adv. Drug Deliv. Rev..

[91] Ong, C. S. et al. Biomaterial-free three-dimensional bioprinting of cardiac tissue using human induced pluripotent stem cell derived cardiomyocytes, *Sci. Rep.-Uk* **7**, 4566 (2017). 10.1038/s41598-017-05018-4 PMC5496874 28676704

[92] Liu Y (2022). hESCs-derived early vascular cell spheroids for cardiac tissue vascular engineering and myocardial infarction treatment. Adv. Sci. (Weinh.).

[93] Noguchi R (2016). Development of a three-dimensional pre-vascularized scaffold-free contractile cardiac patch for treating heart disease. J. Heart Lung Transpl..

[94] Kawaguchi S (2021). Intramyocardial transplantation of human iPS cell-derived cardiac spheroids improves cardiac function in heart failure animals. JACC Basic Transl. Sci..

[95] Lee JH, Han YS, Lee SH (2016). Long-duration three-dimensional spheroid culture promotes angiogenic activities of adipose-derived mesenchymal stem cells. Biomol. Ther..

[96] Lee J (2020). Human adipose-derived stem cell spheroids incorporating platelet-derived growth factor (PDGF) and bio-minerals for vascularized bone tissue engineering. Biomaterials.

[97] Kim EM (2019). Fabrication of core-shell spheroids as building blocks for engineering 3D complex vascularized tissue. Acta Biomater..

[98] Kim SK (2016). Combination of three angiogenic growth factors has synergistic effects on sprouting of endothelial cell/mesenchymal stem cell-based spheroids in a 3D matrix. J. Biomed. Mater. Res. B.

[99] Wansleeben C, Barkauskas CE, Rock JR, Hogan BLM (2015). Stem cells of the adult lung: their development and role in homeostasis, regeneration, and disease (vol 2, pg 131, 2013). Wires Dev. Biol..

[100] Henry E (2015). Adult lung spheroid cells contain progenitor cells and mediate regeneration in rodents with bleomycin-induced pulmonary fibrosis. Stem Cells Transl. Med..

[101] Dinh PC (2017). Derivation of therapeutic lung spheroid cells from minimally invasive transbronchial pulmonary biopsies. Respir. Res..

[102] Lazar A (1995). Formation of porcine hepatocyte spheroids for use in a bioartificial liver. Cell Transplant..

[103] Kizawa H, Nagao E, Shimamura M, Zhang G, Torii H (2017). Scaffold-free 3D bio-printed human liver tissue stably maintains metabolic functions useful for drug discovery. Biochem. Biophys. Rep..

[104] Bierwolf J (2016). Primary human hepatocytes repopulate livers of mice after in vitro culturing and lentiviral-mediated gene transfer. Tissue Eng. Part A.

[105] Regmi S (2019). Intraportally delivered stem cell spheroids localize in the liver and protect hepatocytes against GalN/LPS-induced fulminant hepatic toxicity. Stem Cell Res. Ther..

[106] Yanagi, Y. et al. In vivo and ex vivo methods of growing a liver bud through tissue connection. *Sci. Rep.-Uk* **7**, 14085 (2017). 10.1038/s41598-017-14542-2 PMC5658340 29074999

[107] Song Y (2021). Identification of hepatic fibrosis inhibitors through morphometry analysis of a hepatic multicellular spheroids model. Sci. Rep..

[108] Mannaerts I (2020). The fibrotic response of primary liver spheroids recapitulates in vivo hepatic stellate cell activation. Biomaterials.

[109] Li F, Cao L, Parikh S, Zuo R (2020). Three-dimensional spheroids with primary human liver cells and differential roles of Kupffer cells in drug-induced liver injury. J. Pharm. Sci..

[110] Ray K (2014). Intestinal tract. Patient-derived intestinal spheroids-culturing the gut. Nat. Rev. Gastroenterol. Hepatol..

[111] VanDussen KL (2015). Development of an enhanced human gastrointestinal epithelial culture system to facilitate patient-based assays. Gut.

[112] Chia SL, Tay CY, Setyawati MI, Leong DT (2015). Biomimicry 3D gastrointestinal spheroid platform for the assessment of toxicity and inflammatory effects of zinc oxide nanoparticles. Small.

[113] Khalil, H. A. et al. Intestinal epithelial replacement by transplantation of cultured murine and human cells into the small intestine. *PLoS ONE* **14**, e0216326 (2019). 10.1371/journal.pone.0216326 PMC6544206 31150401

[114] Mikos AG, Papadaki MG, Kouvroukoglou S, Ishaug SL, Thomson RC (1994). Mini-review: islet transplantation to create a bioartificial pancreas. Biotechnol. Bioeng..

[115] Shapiro AMJ (2000). Islet transplantation in seven patients with type 1 diabetes mellitus using a glucocorticoid-free immunosuppressive regimen. N. Engl. J. Med..

[116] Dionne KE, Colton CK, Yarmush ML (1993). Effect of hypoxia on insulin secretion by isolated rat and canine islets of Langerhans. Diabetes.

[117] Cooper-Jones, B. & Ford, C. in *CADTH Issues in Emerging Health Technologies* 1–9 (Canadian Agency for Drugs and Technologies in Health, 2016). 29369575

[118] Clayton HA, London NJ, Colloby PS, Bell PR, James RF (1991). The effect of capsule composition on the biocompatibility of alginate-poly-l-lysine capsules. J. Microencapsul..

[119] Cruise GM (1999). In vitro and in vivo performance of porcine islets encapsulated in interfacially photopolymerized poly(ethylene glycol) diacrylate membranes. Cell Transpl..

[120] Liao SW (2013). Maintaining functional islets through encapsulation in an injectable saccharide-peptide hydrogel. Biomaterials.

[121] Lee BR (2012). In situ formation and collagen-alginate composite encapsulation of pancreatic islet spheroids. Biomaterials.

[122] Takaichi S (2022). Three-dimensional vascularized β-cell spheroid tissue derived from human induced pluripotent stem cells for subcutaneous islet transplantation in a mouse model of type 1 diabetes. Transplantation.

[123] Park YS (2016). Scaffold-free parathyroid tissue engineering using tonsil-derived mesenchymal stem cells. Acta Biomater..

[124] Vizoso, F. J., Eiro, N., Cid, S., Schneider, J. & Perez-Fernandez, R., Mesenchymal stem cell secretome: toward cell-free therapeutic strategies in regenerative medicine. *Int. J. Mol. Sci*. **18**, 1852 (2017). 10.3390/ijms18091852 PMC5618501 28841158

[125] Bonewald L (2019). Use it or lose it to age: a review of bone and muscle communication. Bone.

[126] Arufe MC (2011). Analysis of the chondrogenic potential and secretome of mesenchymal stem cells derived from human umbilical cord stroma. Stem Cells Dev..

[127] Miranda JP (2019). The secretome derived from 3D-cultured umbilical cord tissue MSCs counteracts manifestations typifying rheumatoid arthritis. Front. Immunol..

[128] Waters R (2016). Stem cell secretome-rich nanoclay hydrogel: a dual action therapy for cardiovascular regeneration. Nanoscale.

[129] Waters R (2018). Stem cell-inspired secretome-rich injectable hydrogel to repair injured cardiac tissue. Acta Biomater..

[130] Costa EC (2016). 3D tumor spheroids: an overview on the tools and techniques used for their analysis. Biotechnol. Adv..

[131] Wiercinska E (2011). The TGF-β/Smad pathway induces breast cancer cell invasion through the up-regulation of matrix metalloproteinase 2 and 9 in a spheroid invasion model system. Breast Cancer Res. Treat..

[132] Markovitz-Bishitz Y (2010). A polymer microstructure array for the formation, culturing, and high throughput drug screening of breast cancer spheroids. Biomaterials.

[133] Condello S (2015). β-Catenin-regulated ALDH1A1 is a target in ovarian cancer spheroids. Oncogene.

[134] Raghavan S (2015). Formation of stable small cell number three-dimensional ovarian cancer spheroids using hanging drop arrays for preclinical drug sensitivity assays. Gynecol. Oncol..

[135] Aydin O, Vlaisavljevich E, Yuksel Durmaz Y, Xu Z, ElSayed MEH (2016). Noninvasive ablation of prostate cancer spheroids using acoustically-activated nanodroplets. Mol. Pharm..

[136] Miyoshi H (2018). An improved method for culturing patient-derived colorectal cancer spheroids. Oncotarget.

[137] Hortelão AC, Carrascosa R, Murillo-Cremaes N, Patiño T, Sánchez S (2019). Targeting 3D bladder cancer spheroids with urease-powered nanomotors. ACS Nano.

[138] Carduner L (2014). Cell cycle arrest or survival signaling through αv integrins, activation of PKC and ERK1/2 lead to anoikis resistance of ovarian cancer spheroids. Exp. Cell Res..

[139] Riwaldt S (2016). Pathways regulating spheroid formation of human follicular thyroid cancer cells under simulated microgravity conditions: a genetic approach. Int. J. Mol. Sci..

[140] Piccinini F, Tesei A, Arienti C, Bevilacqua A (2015). Cancer multicellular spheroids: volume assessment from a single 2D projection. Comput. Methods Prog. Biomed..

[141] Aparicio S, Hidalgo M, Kung AL (2015). Examining the utility of patient-derived xenograft mouse models. Nat. Rev. Cancer.

[142] Däster S (2017). Induction of hypoxia and necrosis in multicellular tumor spheroids is associated with resistance to chemotherapy treatment. Oncotarget.

[143] Mehta G, Hsiao AY, Ingram M, Luker GD, Takayama S (2012). Opportunities and challenges for use of tumor spheroids as models to test drug delivery and efficacy. J. Control. Release.

[144] Millard M (2017). Drug delivery to solid tumors: the predictive value of the multicellular tumor spheroid model for nanomedicine screening. Int. J. Nanomed..

[145] Gencoglu MF (2018). Comparative study of multicellular tumor spheroid formation methods and implications for drug screening. ACS Biomater. Sci. Eng..

[146] Freeman FE, Burdis R, Mahon OR, Kelly DJ, Artzi N (2022). A spheroid model of early and late-stage osteosarcoma mimicking the divergent relationship between tumor elimination and bone regeneration. Adv. Healthc. Mater..

[147] Fang Y, Eglen RM (2017). Three-dimensional cell cultures in drug discovery and development. SLAS Discov..

[148] Xu J, Patton D, Jackson SK, Purcell WM (2013). In-vitro maintenance and functionality of primary renal tubules and their application in the study of relative renal toxicity of nephrotoxic drugs. J. Pharmacol. Toxicol. Methods.

[149] Xu Y, Shi TP, Xu AX, Zhang L (2016). 3D spheroid culture enhances survival and therapeutic capacities of MSCs injected into ischemic kidney. J. Cell. Mol. Med..

[150] Tostoes RM (2012). Human liver cell spheroids in extended perfusion bioreactor culture for repeated-dose drug testing. Hepatology.

[151] Bhise NS (2016). A liver-on-a-chip platform with bioprinted hepatic spheroids. Biofabrication.

[152] Mukundan, S. et al. In vitro miniaturized tuberculosis spheroid model. *Biomedicines* **9**, 1209 (2021). 10.3390/biomedicines9091209 PMC8470281 34572395

[153] Li W (2003). Angiotensin-converting enzyme 2 is a functional receptor for the SARS coronavirus. Nature.

[154] Wang S (2021). AXL is a candidate receptor for SARS-CoV-2 that promotes infection of pulmonary and bronchial epithelial cells. Cell Res.

[155] Bugatti, A. et al. SARS-CoV-2 infects human ACE2-negative endothelial cells through an α_v_β_3_ integrin-mediated endocytosis even in the presence of vaccine-elicited neutralizing antibodies. *Viruses* **14**, 705 (2022). 10.3390/v14040705 PMC9032829 35458435

[156] Li Z (2021). Cell-mimicking nanodecoys neutralize SARS-CoV-2 and mitigate lung injury in a non-human primate model of COVID-19. Nat. Nanotechnol..

[157] White ES (2015). Lung extracellular matrix and fibroblast function. Ann. Am. Thorac. Soc..

[158] Hettle R (2017). The assessment and appraisal of regenerative medicines and cell therapy products: an exploration of methods for review, economic evaluation and appraisal. Health Technol. Assess..

[159] Kim M, Yun H-W, Park DY, Choi BH, Min B-H (2018). Three-dimensional spheroid culture increases exosome secretion from mesenchymal stem cells. Tissue Eng. Regen. Med..

[160] Cha JM (2018). Efficient scalable production of therapeutic microvesicles derived from human mesenchymal stem cells. Sci. Rep.-Uk.

